# Cross-cultural adaptation and psychometric assessment of a Brazilian-Portuguese version of the Resident Questionnaire

**DOI:** 10.1371/journal.pone.0203531

**Published:** 2018-09-04

**Authors:** Karina Pereira-Lima, Ana Paula Casagrande Silva-Rodrigues, Flávia Andressa Farnocchi Marucci, Flávia de Lima Osório, José Alexandre Crippa, Sonia Regina Loureiro

**Affiliations:** Department of Neuroscience and Behavior, University of São Paulo, Ribeirão Preto, São Paulo, Brazil; University of Botswana Faculty of Medicine, BOTSWANA

## Abstract

**Background:**

Despite the general agreement regarding the central role of the clinical learning environment in graduate medical education, its assessment remains challenging owing to the lack of available standardized measures. We report on the cross-cultural adaptation and psychometric assessment of the Brazilian-Portuguese version of Seelig’s Resident Questionnaire.

**Methods:**

The present study was performed in two steps. First, a cross-cultural translation and adaptation of the Resident Questionnaire was conducted through multiple translations, synthesis of versions, back-translation, content validation, and face validation. Subsequently, a sample of 288 (72%) resident physicians enrolled in 40 residency programs at a Brazilian university hospital completed the following measures: 1) the Brazilian-Portuguese version of the Resident Questionnaire (for factor analysis and to determine internal consistency, reliability, and validity); 2) three existing, validated psychometric measures (to determine convergent and divergent validity); and 3) a self-report questionnaire.

**Results:**

Confirmatory factor analysis results provided support for the three-dimensional model of the Resident Questionnaire in use on a sample of Brazilian resident physicians, having been previously verified for use in American samples. All three factors (emotional distress, learning environment satisfaction, and workload satisfaction) verified in the confirmatory factor analysis showed good internal consistency (α > .80), reliability (Raykov’s rho > .80), and correlations in the expected directions and magnitude with measures of depressive symptoms, duty hours, organizational conditions, and emotional exhaustion.

**Conclusions:**

This study is the first to adapt a measure of the clinical learning environment of residency programs into Brazilian Portuguese. Our findings suggest that the adapted version of the Resident Questionnaire is valid and reliable for assessing Brazilian residency programs. This free, easy-access, and fast-application instrument may be a useful standardized measure for research and educational purposes concerning the clinical learning environments of resident physicians.

## Introduction

The significance of the clinical learning environment in resident physicians’ performance, well-being, and patient care has been a topic of increasing debate in recent literature [1–4]. Although the clinical learning environment is likely to play a central role in graduate medical education, its assessment remains challenging, owing to the lack of available standardized measures [5].

Among the standardized measures for evaluating the clinical learning environments of resident physicians, the Resident Questionnaire (RQ) has been demonstrated to be a valid measure for assessing residents’ perceptions of critical aspects of their programs [6]. Since its development in 1993 [7] and subsequent revision and validation in 1995 [6], the aspects measured by the RQ have been associated with mental health, education, and satisfaction of resident physicians and fellows from different programs in the United States [6, 8–11].

In the original validation study of the RQ, residents were asked to indicate whether they agreed with 33 statements using a self-report Likert scale ranging from *1 = Strongly Disagree* to *5 = Strongly Agree* [6]. The results identified 28 valid items categorized into the following three correlated first-order factors: emotional distress (11 items, α = .89), workload satisfaction (8 items, α = .85), and learning environment satisfaction (9 items, α = .82). The correlation analysis in the original study verified strong correlations between emotional distress and workload satisfaction (r = -.71). Less robust correlations were found between emotional distress and learning environment satisfaction (r = -.47), and between workload satisfaction and learning environment satisfaction (r = .55). These three dimensions evaluated by the RQ have been shown to be valid for the assessment of different aspects of residents’ perceptions of their programs [6].

Although other measures of the clinical environment of resident physicians have been developed in recent years, most do not present strong evidence of validity [5]. Others assess numerous factors related to the residency context/legislation of a specific country [12, 13], or are long [13, 14], which could impair their use in large surveys on resident physicians. In addition, besides its assessment of residents’ satisfaction with their workload and learning environment, the RQ has the advantage of including a measure of residents’ emotional distress. This could be useful for identifying trainees at risk of developing stress-related problems.

Herein, we set out to describe the process of the cross-cultural adaptation and psychometric assessment of a Brazilian-Portuguese version of the RQ. Our study uses a sample of resident physicians enrolled in 40 residency programs at a Brazilian university hospital. Our specific goals were as follows: a) to perform a cross-cultural translation and adaptation of the RQ to Brazilian Portuguese; b) to assess the content and face validity of this Brazilian-Portuguese version; c) to examine the factor structure of the Brazilian-Portuguese RQ; d) to assess the internal consistency and reliability of the Brazilian-Portuguese RQ; and e) to evaluate the convergent and divergent validity of the Brazilian-Portuguese RQ, with measures of both positive and negative organizational characteristics, depressive symptoms, and emotional exhaustion.

## Methods

After obtaining ethical approval from the Institutional Review Board, the present study was conducted using the following two steps: 1) cross-cultural translation and adaptation of the RQ into Brazilian Portuguese and 2) psychometric assessment of the validity and reliability of the Brazilian-Portuguese RQ. All participants provided informed consent.

### Step 1: Cross-cultural translation and adaptation

The RQ was translated and adapted into Brazilian Portuguese in accordance with standardized technical recommendations [15], as described below.

First, the instrument was independently translated by two bilingual psychiatry residents and two professional translators with more than five years of experience (one Brazilian and one American). These four versions were synthesized by an experienced psychiatrist and an experienced psychologist into a single Brazilian-Portuguese version. This version was then back-translated by a professional American translator.

For content validation, both forward- and back-translation were evaluated by an expert committee comprising one bilingual psychiatrist and two bilingual clinical psychologists, each with more than 10 years of experience in medical residency education. These experts were asked to evaluate whether the items in the two versions were conceptually similar to the target constructs of the original instrument. The conceptual, semantic, idiomatic, and cultural equivalence of the instructions and each of the 28 items were rated by the experts, who suggested cross-cultural adaptation of three items, as described below.

Since the term, *hospital support services*, is not typically used in the Brazilian health system, the experts suggested adding examples of types of hospital support services in parentheses after the term in the item, “Hospital support services sufficiently help me care for my patients.” Similarly, the experts suggested changing the term, *conferences*, to “*reuniões clínicas*” (translation: clinical meetings) in the item, “The scheduled conferences are generally a valuable learning experience,” since this term would be more appropriate for the context in which theoretical and applied subjects are taught and discussed in Brazilian residency programs. Finally, considering differences in the demands of the various specialties included in this instrument undergoing adaptation, the experts suggested adapting the item, “The average number of workups on call days is reasonable,” to “*O número médio de chamados (pedidos de exames*, *bips*, *urgências*, *intercorrências) em dias de plantão é razoável*” (translation: The average number of calls [workups, bips, urgencies, intercurrences] on call days is reasonable).

For face validation, pilot testing was conducted with a small number (n = 7) of residents from medical and surgical specialties (internal medicine, general surgery, ophthalmology, psychiatry, and radiology) to determine semantic understanding of the instructions and items of the Brazilian-Portuguese version of the RQ. Residents were asked to read and then rephrase all sentences in the instrument. Since they did not demonstrate any difficulty understanding any of the RQ items, this step was considered to be the conclusion of the translation and adaptation of the RQ into Brazilian Portuguese.

### Step 2: Psychometric assessment of the validity and reliability of the Brazilian-Portuguese version of the RQ

#### Data collection

A total of 400 medical residents of both sexes with varying years of residency (1^st^ to 5^th^) and specialties at a Brazilian university hospital were invited to participate in the present study between August and December of 2016. The referral hospital is a public university-based institution located in a medium-sized (682,302 people) inner state city in the state of São Paulo, Brazil. Residents were eligible to participate in the study if they were Brazilian or foreign nationals who had resided in Brazil for at least two years. Residents were recruited at their workplaces and those who agreed to participate signed informed consent forms and were asked to complete the following self-report instruments: the Brazilian-Portuguese version of the RQ to obtain data for confirmatory factor analysis (CFA) and to determine the measure’s internal consistency, reliability, and validity; the Patient Health Questionnaire-9 (PHQ-9) [16]; the Burnout Syndrome Inventory (BSI) [17]; a question about weekly duty hours to determine the RQ’s convergent and divergent validity; and a self-report questionnaire on demographic variables including sex, age, residency program, and year of postgraduate study.

The PHQ-9 is a component of the Primary Care Evaluation of Mental Health Disorders inventory comprising nine self-report items designed to screen respondents for depressive symptoms [16]. The instrument has good psychometric properties, as demonstrated in validation studies with primary healthcare patients and the general population [16, 18, 19]. The Positive Organizational Conditions (BSI-POC), Negative Organizational Conditions (BSI-NOC), and Emotional Exhaustion (BSI-EE) scales of the BSI were used in the present study. The full BSI is a Brazilian instrument consisting of 35 items categorized into two parts (16 and 19 items, respectively) [17]. The first part assesses organizational factors reported in literature as triggers or modulators of occupational stress and, consequently, burnout. The second part assesses burnout syndrome through four dimensions [17]. Validation studies with participants from various professional fields, including health care, have demonstrated the psychometric adequacy of the BSI [17].

#### Descriptive analysis

After verifying that data on all the variables were normally distributed, using Kolmogorov-Smirnov normality tests, we conducted descriptive analysis (mean, standard deviation, frequency, percentage) to characterize the demographic and academic profiles of the study sample.

#### Confirmatory factor analysis (CFA)

We conducted CFA to evaluate whether the three-dimensional model of the RQ proposed for assessing American residency programs fits the Brazilian residency context. To account for the categorical characteristics of the RQ items, we performed CFA using the Weighted Least Squares Means and Variance Adjusted estimation method in Mplus 7.11. Model fit was evaluated through the Comparative Fit Index (CFI), Tucker-Lewis Index (TLI), and the Root Mean Square Error of Approximation (RMSEA) with a 90% confidence interval. CFI and TLI values higher than .90 and RMSEA values lower than .08 represent acceptable fit, and CFI and TLI values higher than .95 and RMSEA values lower than .05 represent excellent fit [20, 21]. Items were accepted as part of a particular scale if a factor loading of at least .45 was obtained [22].

#### Reliability analysis

The reliability of the Brazilian-Portuguese version of the RQ was assessed through internal consistency analysis using Cronbach’s alpha, and composite reliability using Raykov’s rho coefficient. Alpha values higher than .70 and rho values higher than .80 indicate good reliability [23, 24].

#### Convergent and divergent validation

Convergent and divergent validity were assessed through Pearson’s correlations for the RQ scales and measures of the BSI-POC, BSI-NOC, BSI-EE, PHQ-9 depressive symptoms, and average duty hours per week.

## Results

### Representativeness of the sample

A total of 288 Brazilian medical residents from 40 residency programs were included in the present study (response rate = 72%). Participants’ demographic and academic characteristics are presented in Table 1.

**Table 1:** Participants’ demographic and academic characteristics.

Sex, Frequency (%)	
Female	146 (50.7%)
Male	142 (49.3%)
**Age, Mean (Standard Deviation)**	28.0 (2.6)
**Years, Frequency (%)**	
Initial (1^st^ to 2^nd^)	200 (69.4%)
Final (3^rd^ to 5^th^)	88 (30.6%)
**Specialty field, Frequency (%)**	
Medical	178 (61.8%)
Surgical	110 (38.2%)
**Duty hours per week, Mean (Standard Deviation)**	65.0 (17.0)

### CFA, internal consistency, and reliability

The CFA of the Brazilian-Portuguese RQ showed an acceptable fit of the model proposed for American Programs in the Brazilian residency context (RMSEA 0.073, 90% CI 0.067–0.079; CFI .938, TLI .932). Pearson’s correlation analysis revealed significant correlations between emotional distress and workload satisfaction (r = -.542, p < .001), emotional distress and learning environment satisfaction (r = -.372, p < .001), and workload satisfaction and learning environment satisfaction (r = .408, p < .001). Similarly, all scales of the Brazilian-Portuguese RQ showed good internal consistency and reliability (α > .70, rho > .80).

Table 2 presents Cronbach’s alpha value and the Raykov’s composite reliability rho coefficient for each dimension of the Brazilian-Portuguese RQ, as well as the standardized loadings, R^2^, and error variance for each item of the instrument.

**Table 2:** CFA, internal consistency, and reliability of the Brazilian-Portuguese RQ.

DIMENSION/ITEM	Factor loading	R^2^	Error
***Emotional distress* (*11 items*, α = .923, rho = .944)**			
I often have feelings of frustration	.812	.659	.341
I generally enjoy life (reverse-scored)	.702	.493	.507
I frequently feel angry about things that happen at work	.733	.537	.463
I often feel "stressed out"	.844	.712	.288
I sometimes feel like a failure	.706	.498	.502
I am frequently required to take care of patients that I am not sufficiently experienced to handle	.675	.456	.544
I often feel tired	.819	.671	.329
I sometimes have emotional outbursts that I later feel sorry about	.694	.482	.518
I frequently feel overworked	.909	.826	.174
I frequently feel depressed	.821	.674	.326
I find that I become irritated easily	.818	.669	.331
***Workload satisfaction* (*8 items*, α = .891, rho = .916)**			
Time demands are reasonable and allow me to get my work done	.821	.674	.326
Hospital support services (examples: logistic, cleaning, auxiliary workers, technicians) are sufficient to help me care for my patients	.689	.475	.525
The call schedule is too heavy (reverse-scored)	.667	.445	.555
The caseload in this program is about right	.693	.480	.520
I rarely have time to read (reverse-scored)	.715	.511	.489
The average number of calls (workups, bips, urgencies, intercurrences) on call days is reasonable	.778	.605	.395
There is enough clerical and administrative support provided by the program	.838	.702	.298
The workload in this program is generally excessive (reverse- scored)	.866	.750	.250
***Learning environment satisfaction* (*9 items*, α = .870, rho = .895)**			
I get timely and appropriate feedback from faculty	.730	.533	.467
Clinical meetings are generally a valuable learning experience	.603	.364	.646
Inpatient ward rotations are generally a good learning experience	.611	.373	.627
I have received sufficient counseling from faculty to help with career planning	.773	.598	.402
The degree of responsibility that I have for the care of patients is appropriate	.607	.368	.632
Full‐time faculty members contribute to a great extent to the teaching that I have received	.835	.697	.303
I generally feel that other residents are helpful and "do their fair share"	.775	.601	.399
I have enough personal support from faculty	.633	.401	.599
I receive enough instruction on what is expected of me at each level of my training	.696	.484	.516

α: Cronbach’s alpha; rho: Raykov’s rho

### Convergent and divergent validation

Table 3 presents the correlation coefficients for the Brazilian-Portuguese RQ and related measures.

**Table 3:** Convergent and divergent validity of the RQ with measures of depressive symptoms, organizational conditions, and duty hours.

*RQ dimension*	*PHQ-9 score*	*BSI–POC*	*BSI–NOC*	*BSI–EE*	*Duty hours*
**Emotional distress**^**a**^	r = .688 p < .001	r = -.512 p < .001	r = .573 p < 001	r = .650 p < .001	r = .269 p < .001
**Workload satisfaction**^**b**^	r = -.424 p < .001	r = .481 p < .001	r = -.529 p < .001	r = -.409 p < .001	r = -.390 p < .001
**Learning environment satisfaction**^**b**^	r = -.240 p < .001	r = .685 p < .001	r = -.497 p < .001	r = -.278 p < .001	r = -.105 p = .076

^a^ Higher scores indicate higher levels of emotional distress

^b^ Higher scores indicate higher satisfaction

## Discussion

The present study entailed a cross-cultural translation and adaptation of the RQ into Brazilian Portuguese and evaluation of the psychometric adequacy of this instrument using a sample of medical residents enrolled in 40 residency programs at a Brazilian university hospital. Our results demonstrate the adequacy of the original RQ model [6] for the evaluation of Brazilian residency programs, as well as the instrument’s good validity and reliability for use on this sample of Brazilian resident physicians.

Besides confirming the factorial structure identified in the original validation study of the RQ, the relationships between the three dimensions of the Brazilian-Portuguese version were comparable to those identified in the original study [6]. Significant correlations were found between all dimensions of the instrument. In addition, similar to the results of the original study [6], the RQ-Emotional Distress, RQ-Workload Satisfaction, and RQ-Learning Environment Satisfaction scales have shown good internal consistency and reliability in the Brazilian-Portuguese version of Seelig’s RQ.

In line with the results of previous studies using the RQ on American samples [6, 7], our results showed high correlations of measures of depressive symptoms and emotional exhaustion with the RQ–Emotional Distress scale. The results also showed moderate and weak correlations of these measures with the RQ-Workload and RQ-Learning Environment Satisfaction scales. The convergent validity analysis showed significant correlations in the expected directions and magnitude for all dimensions of the RQ with positive (BSI-POC) and negative (BSI-NOC) organizational conditions assessed by the BSI. This demonstrates the validity of the Brazilian-Portuguese RQ for assessing program-related aspects of residents’ clinical learning environment. In addition, the significant moderate correlations between the Workload Satisfaction Scale and average duty hours per week demonstrated that, although related, these are distinct constructs.

With regard to the strengths of the study, first, standardized procedures for cross-cultural translation and adaptation were used [15]. Second, the study assessed residents enrolled in 40 residency programs in medical and surgical specialties. Third, the study demonstrated content and face validity, adequate factor structure fit, good reliability, and convergent and divergent validity of the Brazilian-Portuguese RQ with measures of organizational conditions, exhaustion, depressive symptoms, and duty hours. The limitations include the assessment of residents from one university-based institution in a medium-sized inner state city in São Paulo, and the lack of test-retest reliability. In addition, although previous research has shown that self-reported duty hours match well with electronic records [25], the average number of duty hours per week may have been misreported. There is a need for further studies on residents from different types of institutions (public/private, university/community-based) and regions, and those assessing test-retest reliability and both the discriminative and predictive validity of the Brazilian-Portuguese RQ. These would enable better exploration of the psychometric adequacy of this version of the instrument.

In summary, this cross-cultural adaptation and validation study showed the Brazilian-Portuguese version of the RQ to be a valid and reliable measure for assessing different aspects of residents’ perceptions of the workload, learning environment, and emotional distress related to their programs. This free, easy-access, and fast-application instrument may be a useful standardized measure for research and educational purposes concerning the clinical learning environment of resident physicians.

## Supporting information

S1 File Brazilian-Portuguese version of the Resident Questionnaire. (PDF)

S2 File Calculation of results. (PDF)

S3 File Dataset. (XLSX)
