# Supplementary material for: Cross-cultural adaptation and psychometric assessment of a Brazilian-Portuguese version of the Resident Questionnaire
Source: PLoS One. 2018 Sep 4;13(9):e0203531. doi: 10.1371/journal.pone.0203531 (PMC6122823; doi:10.1371/journal.pone.0203531)
Supplement: S1 File — (PDF) [file pone.0203531.s001.pdf]

QUESTIONÁRIO PARA RESIDENTES

Por favor, preencha o seguinte questionário que tem por objetivo identificar informações que podem ajudar a melhorar os programas de residência médica. Responda a todas as questões abaixo, usando a escala fornecida, circulando a resposta que melhor reflete a sua avaliação.

Discordo fortemente				Concordo fortemente
1	2	3	4	5

**1.** Eu recebo devolutivas *(feedbacks)* apropriadas dos supervisores no momento oportuno.	1	2	3	4	5
**2.** As exigências em relação a prazos para as tarefas são razoáveis e me permitem fazer o meu trabalho.	1	2	3	4	5
**3.** Eu frequentemente me sinto frustrado/a.	1	2	3	4	5
**4.** Os serviços de apoio hospitalar (exemplos: logística, limpeza, auxiliares e técnicos) são suficientes para me ajudar a cuidar de meus pacientes.	1	2	3	4	5
**5.** Eu geralmente aproveito a vida.	1	2	3	4	5
**6.** As reuniões clínicas programadas geralmente são experiências de aprendizagem valiosas.	1	2	3	4	5
**7.** Eu frequentemente me sinto irritado/a com coisas que acontecem no trabalho.	1	2	3	4	5
**8.** A escala de plantões é muito pesada.	1	2	3	4	5
**9.** As rotações de estágio junto a pacientes internados geralmente são uma boa experiência de aprendizagem.	1	2	3	4	5
**10.** Tenho recebido aconselhamento suficiente dos supervisores para ajudar no planejamento de minha carreira.	1	2	3	4	5
**11.** Eu frequentemente me sinto estressado/a.	1	2	3	4	5
**12.** O número de casos atendidos nesse programa de residência está adequado.	1	2	3	4	5
**13.** Às vezes eu me sinto um fracasso.	1	2	3	4	5
**14.** Eu frequentemente sou designado/a a cuidar de pacientes com os quais eu não tenho experiência suficiente para lidar.	1	2	3	4	5
**15.** Eu frequentemente me sinto cansado/a.	1	2	3	4	5
**16.** Às vezes tenho reações emocionais pelas quais me sinto mal posteriormente.	1	2	3	4	5
**17.** Eu frequentemente me sinto esgotado/a.	1	2	3	4	5
**18.** O grau de responsabilidade que tenho pelo cuidado dos pacientes é adequado.	1	2	3	4	5
**19.** Eu raramente tenho tempo para leitura.	1	2	3	4	5
**20.** Os docentes de dedicação exclusiva contribuem em grande parte para os ensinamentos que tenho recebido.	1	2	3	4	5
**21.** O número médio de chamados (pedidos de exames, bips, urgências, intercorrências) em dias de plantão é razoável.	1	2	3	4	5
**22.** Eu geralmente sinto que os outros residentes são prestativos e "fazem sua parte".	1	2	3	4	5
**23.** Eu recebo suficiente apoio personalizado por parte dos supervisores.	1	2	3	4	5
**24.** Eu frequentemente me sinto deprimido/a.	1	2	3	4	5
**25.** O apoio administrativo oferecido pelo programa de residência é suficiente para o exercício de minha função.	1	2	3	4	5
**26.** Eu recebo instrução suficiente sobre o que é esperado de mim em cada etapa do meu treinamento.	1	2	3	4	5
**27.** A quantidade de trabalho nesse programa é geralmente excessiva.	1	2	3	4	5
**28.** Eu acho que estou ficando facilmente irritado/a.	1	2	3	4	5

Instrumento originalmente desenvolvido por Seelig, CB et al. Development and validation of a scaled questionnaire for evaluation of residency programs. Southern Medical Journal. 1995;88(7):745-50. Traduzido, adaptado e validado para o português-brasileiro por Karina Pereira-Lima, Ana Paula Casagrande Silva-Rodrigues, Flávia Andressa Farnocchi Marucci, Flávia de Lima Osório, José Alexandre Crippa e Sonia Regina Loureiro.
