# Supplementary material for: Cross-cultural adaptation and psychometric assessment of a Brazilian-Portuguese version of the Resident Questionnaire
Source: PLoS One. 2018 Sep 4;13(9):e0203531. doi: 10.1371/journal.pone.0203531 (PMC6122823; doi:10.1371/journal.pone.0203531)
Supplement: S2 File — (PDF) [file pone.0203531.s002.pdf]

QUESTIONÁRIO PARA RESIDENTES: CÁLCULO DOS RESULTADOS

(Resident Questionnaire: Calculation of results)

Sofrimento emocional (SE) *(Emotional Distress - ED)*

Cálculo *(calculation)*: **SE = (Q03 + [6 – Q05] + Q07 + Q11 + Q13 + Q14 + Q15 + Q16 + Q17 + Q24 + Q28) ÷ 11**

Interpretação *(interpretation)*: quanto maior o escore, maior o nível de sofrimento emocional.

Q03 - Eu frequentemente me sinto frustrado/a.
Q05 - Eu geralmente aproveito a vida.
Q07 - Eu frequentemente me sinto irritado/a com coisas que acontecem no trabalho.
Q11 - Eu frequentemente me sinto estressado/a.
Q13 - Às vezes eu me sinto um fracasso.
Q14 - Eu frequentemente sou designado/a a cuidar de pacientes com os quais eu não tenho experiência suficiente para lidar.
Q15 - Eu frequentemente me sinto cansado/a.
Q16 - Às vezes tenho reações emocionais pelas quais me sinto mal posteriormente.
Q17 - Eu frequentemente me sinto esgotado/a.
Q24 - Eu frequentemente me sinto deprimido/a.
Q28 - Eu acho que estou ficando facilmente irritado/a.

Satisfação com a carga de trabalho (SCT) *(Workload satisfaction - WS)*

Cálculo *(calculation)*: **SCT = (Q02 + Q04 + [6 – Q08] + Q12 + [6 – Q19] + Q21 + Q25 + [6 – Q27]) ÷ 8**

Interpretação *(interpretation)*: quanto maior o escore, maior o nível de satisfação com a carga de trabalho.

Q02 - As exigências em relação a prazos para as tarefas são razoáveis e me permitem fazer o meu trabalho.
Q04 - Os serviços de apoio hospitalar (exemplos: logística, limpeza, auxiliares e técnicos) são suficientes para me ajudar a cuidar de meus pacientes.
Q08 - A escala de plantões é muito pesada.
Q12 - O número de casos atendidos nesse programa de residência está adequado.
Q19 - Eu raramente tenho tempo para leitura.
Q21 - O número médio de chamados (pedidos de exames, bips, urgências, intercorrências) em dias de plantão é razoável.
Q25 - O apoio administrativo oferecido pelo programa de residência é suficiente para o exercício de minha função.
Q27 - A quantidade de trabalho nesse programa é geralmente excessiva.

Satisfação com o ambiente de aprendizagem (SAA) *(Learning environment satisfaction - LES)*

Cálculo *(calculation)*: **SAA = (Q01 + Q06 + Q09 + Q10 + Q18 + Q20 + Q22 + Q23 + Q26) ÷ 9**

Interpretação *(interpretation)*: quanto maior o escore, maior o nível de satisfação com o ambiente de aprendizagem.

Q01 - Eu recebo devolutivas (*feedbacks*) apropriadas dos supervisores no momento oportuno.
Q06 - As reuniões clínicas programadas geralmente são experiências de aprendizagem valiosas.
Q09 - As rotações de estágio junto a pacientes internados geralmente são uma boa experiência de aprendizagem.
Q10 - Tenho recebido aconselhamento suficiente dos supervisores para ajudar no planejamento de minha carreira.
Q18 - O grau de responsabilidade que tenho pelo cuidado dos pacientes é adequado.
Q20 - Os docentes de dedicação exclusiva contribuem em grande parte para os ensinamentos que tenho recebido.
Q22 - Eu geralmente sinto que os outros residentes são prestativos e “fazem sua parte”.
Q23 - Eu recebo suficiente apoio personalizado por parte dos supervisores.
Q26 - Eu recebo instrução suficiente sobre o que é esperado de mim em cada etapa do meu treinamento.
